# PRASH: A Framework for Privacy Risk Analysis of Smart Homes

**DOI:** 10.3390/s21196399

**Published:** 2021-09-25

**Authors:** Joseph Bugeja, Andreas Jacobsson, Paul Davidsson

**Affiliations:** Internet of Things and People Research Center, Department of Computer Science and Media Technology, Malmö University, 21579 Malmö, Sweden; andreas.jacobsson@mau.se (A.J.); paul.davidsson@mau.se (P.D.)

**Keywords:** smart home, IoT, privacy, risk analysis, system model, threat model, privacy metrics, attack taxonomy

## Abstract

Smart homes promise to improve the quality of life of residents. However, they collect vasts amounts of personal and sensitive data, making privacy protection critically important. We propose a framework, called PRASH, for modeling and analyzing the privacy risks of smart homes. It is composed of three modules: a system model, a threat model, and a set of privacy metrics, which together are used for calculating the privacy risk exposure of a smart home system. By representing a smart home through a formal specification, PRASH allows for early identification of threats, better planning for risk management scenarios, and mitigation of potential impacts caused by attacks before they compromise the lives of residents. To demonstrate the capabilities of PRASH, an executable version of the smart home system configuration was generated using the proposed formal specification, which was then analyzed to find potential attack paths while also mitigating the impacts of those attacks. Thereby, we add important contributions to the body of knowledge on the mitigations of threat agents violating the privacy of users in their homes. Overall, the use of PRASH will help residents to preserve their right to privacy in the face of the emerging challenges affecting smart homes.

## 1. Introduction

In recent years, the pervasiveness of Internet of Things (IoT) technologies has contributed to transforming the home into a smart home. The smart home is one of the most well-known IoT applications, in which heterogeneous devices ranging from smart speakers to electronic door locks are connected to the home network and controlled remotely through the Internet. These connected devices collect and exchange data with each other and their users using embedded sensors and the Internet, seamlessly merging the physical and digital worlds inside the home. Consequently, these technologies are linked to a variety of benefits, including improved convenience, energy efficiency, enhanced security and safety, and more [1]. The smart home market has seen rapid growth over the past few years. Indeed, the global smart home market was projected to reach approximately $53.45 billion in 2022 [2] with an estimated compound growth of more than 14.5% from 2017 to 2022. This demonstrates the increasing consumer demand and rising adoption of this technology.

As smart homes become more popular, the potential for exploitation by malicious threat agents is likely to increase. Indeed, the growing use of smart home devices in households is accompanied by substantial privacy risks stemming from having data being misused by malicious threat agents [3,4,5]. The home is often described as a person’s castle or sanctuary; a private and protected space. Residents, for example, expect that their intimate conversations, emotions, expressions, family photos, video footage, and daily activities, will remain inside the home and will thus not be shared with unauthorized entities. However, smart home devices challenge this assumption. Various devices are installed with cameras, microphones, location trackers, and may have weak built-in privacy and security measures. This makes them vulnerable to cyber attacks that could expose private data and information about the residents, including children and visitors. Some examples of common attack vectors (i.e., methods used to circumvent the security or privacy of a system) targeting connected devices are vulnerable services, weak authentication, and default configurations [6]. A threat agent may need to only compromise a vulnerability in a single component, such as the smart home gateway, to obtain access to the home area network, and as a result, access household data. It is thus critical to understand the privacy implications of connected devices so that consumers are aware of privacy risks and that these risks can be mitigated without putting the responsibility completely on the users.

Accordingly, we propose a novel contribution to the field of smart homes, specifically a framework called PRASH (Privacy Risk Analysis of Smart Homes), for modeling and analyzing privacy risks of smart homes. PRASH is primarily aimed at researchers and analysts with an interest in smart home systems to help deepen their understanding and reasoning about the privacy concerns affecting such systems. The proposed framework is intended to guide these people in systematically identifying privacy threats and assessing the privacy risks associated with them. For analyzing privacy risks and automating their detection and evaluation, PRASH uses a privacy attack taxonomy, attack trees, and privacy metrics. Given the dynamic and evolving features of smart home technologies, providing automatic methods for conducting risk evaluation is a vital requirement. Existing risk assessment procedures were developed in large part prior to the IoT [7], and as a result, they may not be able to handle the complexity or pervasiveness of smart homes. PRASH was designed to address that need, to raise awareness of risks arising in the smart home. Through the use of quantitative metrics, the framework also helps remove potential subjectivity that might emerge while conducting a manual risk analysis process. This is done through the risk scores, indicating the severity levels of privacy violations, which may be automatically computed through the framework’s algorithm. Overall, the proposed framework contributes to the advancement of risk analysis research for smart homes where privacy enforcement is a critical element, and deepens the understanding of risks introduced when IoT devices are added to private homes.

The rest of the paper is organized as follows. In Section 2, we describe the components of a smart home. Next, in Section 3, we construct a taxonomy of privacy attacks. In Section 4, we present related work on threat modeling, and risk analysis models and frameworks related to the smart home and the IoT. Then, in Section 5, we describe our proposed framework for modeling and analyzing privacy risks of smart homes. The framework’s usefulness is illustrated in Section 6 in a practical use case that instantiated and used an encoded version of the system model. In Section 7, we discuss extensions and limitations of the framework. Finally, in Section 8, we conclude the paper and identify some avenues for future work.

## 2. The Smart Home Components

**Figure 1:**
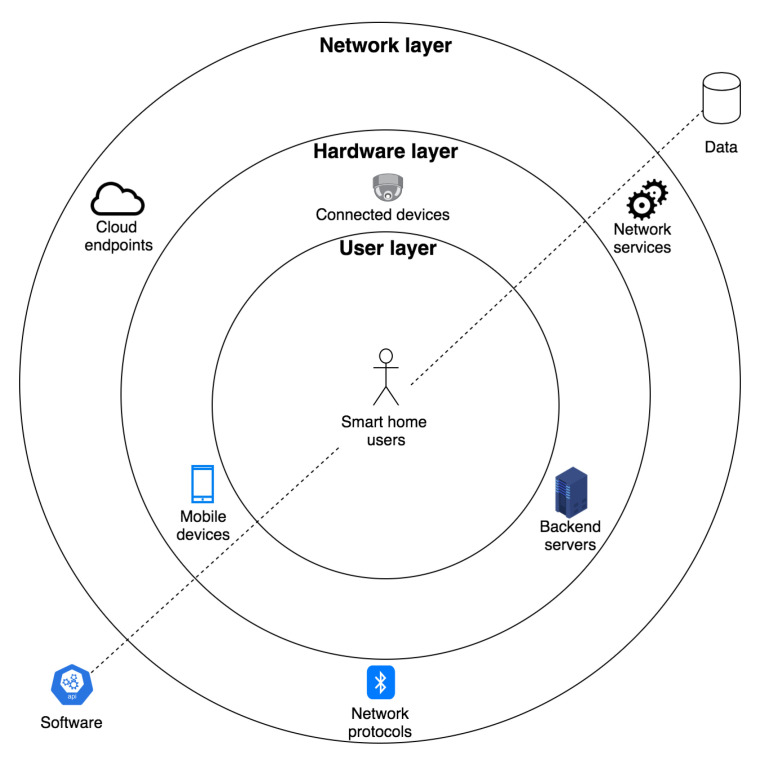
The components of a smart home. At the center are the users, particularly the smart home residents. Users interact with their home via the hardware layer, typically through mobile devices. The network layer is responsible for implementing the communication and providing connectivity between the users and their homes. Data and software represent crosscutting components as data are generated, collected, processed, and exchanged at different layers, and software, which can include machine learning models, is integrated in the different conceptual layers.

Smart homes can be analyzed from the perspective of sociotechnical systems of systems. Sociotechnical systems of systems incorporate human behavior, technology, and policies that influence human behavior by combining the cyber (digital) world with the physical world [8]. Based on the work of Lopez et al. [9] in relation to the analysis of privacy threats in scenarios involving sensing technologies, we consider the user and network as the entities being threatened. Moreover, we also add the hardware as an additional entity that can be threatened directly by privacy attacks. Using this representation, we illustrate the components of a smart home in Figure 1 and describe each conceptual layer. Based on the functional classification of smart home devices we proposed earlier in [10], in Table 1, we summarize the hardware and software capabilities that are supported by smart home devices.

Hardware layer. The physical layer consists of the physical components of the smart home. Components include the connected devices, such as home appliances; networking devices such as routers, switches, and gateways; and sensors that can be attached to different objects. Included here are also mobile devices such as smartphones that are used by end-users to configure and manage the home. Typically, commercial smart homes interconnect devices through a central gateway (hub) device [11]. Gateways connect the local IoT (home area) network to the Internet, oftentimes via a residential router. Commonly, a smart home architecture utilizes a backend architecture, leveraging cloud data centers for processing user data and for rendering its services. At a minimum, devices have firmware, but may also include an operating system and support for services and applications.

Network layer. The network layer connects devices found in the hardware layer and allows for remote control of the home. A core enabler for smart homes is the communication infrastructure and related networking protocols. Various networking technologies such as Zigbee, Wi-Fi, and BLE protocols may be supported by devices found in the hardware layer. The network layer may also incorporate application layer protocols such as MQTT, CoAP, and HTTP. A smart home may also have communication endpoints that interconnect it to systems hosted and managed outside of the physical home environment, e.g., to network storage, file sharing applications, and application programming interfaces (APIs), managed by third-parties.

User layer. The user layer forms or recognizes the human entities. This layer includes the smart home residents and other stakeholders that interact with the home. Typically, the user roles can be grouped into data subject, data controller, and data user [12]. The data subject represents the entity, commonly the homeowner or family, that interacts with the smart home and whose data are processed. The data controller represents the entity, commonly the service provider or manufacturer, who is responsible for processing the data subject’s data. The data user represents the entity, typically working with the data controller, who may access the data subject’s data to provide a service.

The integration and interdependencies of the different cyber and physical components of a smart home result in vulnerabilities that can be compromised through cyber attacks. In the next section, we present an overview of attacks aimed at compromising the privacy of the smart home residents.

## 3. Smart Home Privacy Attacks

Privacy threats affecting IoT systems, and hence also smart homes, are characterized by access, collection, processing, and disclosure of sensitive information in contravention of individuals’ expectations [13,14]. Typically, the goal of a threat agent falls into one or more of three categories: (i) stealing information, e.g., user credentials, (ii) tracking user information, e.g., location, or (iii) taking control of a system, e.g., through malware [15]. For privacy risk analysis, the attacker’s goals are typically focused on (i) and (ii). Leveraging the theory of contextual integrity (CI) [16], we assume that a privacy threat occurs, as a result of a successful attack, when private information is accessed in a way that can be used against the original information norms and control of the individual. Specifically, a privacy attack occurs when the user’s identity is revealed or becomes associated with data that are considered private by the data’s subject.

Using the smart home conceptual layers introduced in Section 2 as a reference framework, we identify privacy attacks targeting the smart home. While there are taxonomies that are centered on privacy, for instance, Solove’s taxonomy [17] that attempts to conceptualize the social and legal aspects of privacy, our taxonomy has different objectives. Specifically, we developed the taxonomy to better understand attacks targeting the home by exploiting vulnerabilities arising from its enabling technologies. Having a taxonomy that is applicable to smart homes is a research gap that the taxonomy fills. The taxonomy also focuses on cyber threats to smart homes already observed in the real world or in controlled experiments, along with potential future vulnerabilities exposed by specific configurations and technology. In the threat model in Section 5.2, the taxonomy was used for constructing attack trees.

While we do not claim that the constructed taxonomy is comprehensive, we conducted a systematic search process for identifying literature related to privacy attacks. Specifically, we queried three key databases, i.e., Google Scholar, Scopus, and Web of Science, using the search terms: (“smarthome” OR “connectedhome” OR “homeautomation”) AND (“privacy”) AND (“risk” OR “threat” OR “attack”). The search terms were used against the titles and abstracts of potential contributions. In the search process, we excluded articles that were: non-English texts, not peer-reviewed, or did not cover (i) and (ii), as described above, as attacker goals. Additionally, we excluded grants, patents, and policy documents from the results. The majority of the literature retrieved was from 2015 to 2020. Work predating that term was sparse and did not consider the IoT and AI features of the contemporary home.

**Figure 2:**
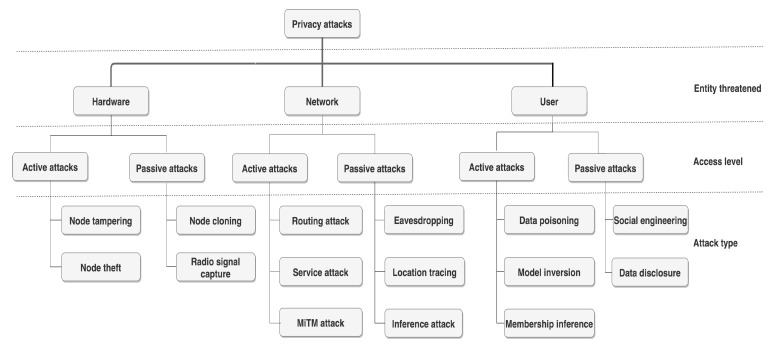
Taxonomy of privacy attacks on the smart home structured, according to the entities they target. Hardware layer attacks target the physical components; network layer attacks target the communication and connectivity; and user layer attacks target the smart home users. Attacks also compromise the software and data that are present across the different conceptual layers of the smart home.

The resulting taxonomy is displayed in Figure 2. While the taxonomy was developed with privacy compromise being the attacker’s main goal, by the nature of privacy it is common that some included attacks, in particular active attacks, also violate the security and safety of individuals. For example, a data poisoning attack (Section 3, item 11 below) may lead to severe, potentially fatal consequences to users, especially when it targets health devices, such as insulin pumps, in certain smart home use cases.

For grouping the retrieved attacks we use the threatened entity as the main classifier and the access level (active attack or passive attack) as a second dimension. The access level dimension was observed as a distinguishing category for privacy attacks in comparison to other attributes, e.g., the attacker’s location. Active attacks occur when a threat agent attempts to alter the system’s resources or affect their operation in order to gain access to information [18]. Passive attacks attempt to learn or make use of information from the system, without affecting the system’s resources [18]. The different attack classes are discussed and exemplified hereunder.

Hardware layer attack. Smart home devices come in various forms, with some being installed at fixed locations (e.g., smart thermostats), some being portable and possibly brought in by guests (e.g., gaming consoles), and others being able to roam around the home (e.g., robotic vacuum cleaners). Regardless of the cases, such devices tend to be subject to hardware compromise.

1. Node tampering: These attacks may range from changing a physical component, e.g., an integrated circuit, in a device, to the installation of a compromised device that may act as a covert spy. An example of a device tampering attack against a smart meter was demonstrated by the researchers in [19]. Tampering may also occur as a direct result of a user borrowing/lending a device for temporary usage.
2. Node theft: By stealing a device [20], a threat agent may increase its time window, for instance, to reverse engineer security systems—including storage—and protocols, with the help of manuals and documentation, to discover data about an individual. This may also include mobile device theft or loss (which has the same effects as theft but is unintentional).
3. Node cloning: Devices, especially tags, can be captured by a threat agent which can then build replicas of them, which look like the original ones. These can then be used to compromise a radio frequency identification (RFID) system by deceiving even the RFID readers [21]. Other devices, for instance, key fobs, that may use near field communication (NFC) as a protocol, may also be subject to cloning. As a result, cloning copies the data found inside a device.
4. Radio signal capture: Radio signals that are emitted by devices, including the residential router, can be used to detect the presence of people and track movements inside the home. For instance, MIT’s RF-Pose project team [22] have trained a neural network to interpret the way radio WiFi signals bounce off a person’s body to learn movements of people. These attacks can potentially be conducted meters away from the home, especially with signal boosters.

Network layer attack. The smart home network may be threatened by different attacks to violate the user’s privacy. Attacks may take advantage of the lack of security measures implemented on the smart home network, for example, a lack of authentication.

5. Routing attack: These attacks occur when the routing table or graph structure of the home network is modified to benefit the malicious intruder. An example of this attack class is a sinkhole attack [23] where an intruder gains control of a node inside a home network to attract all traffic from neighboring nodes. This attack can allow a threat agent access to data flows carrying personal data of individuals.
6. Service attack: Instances of this attack, such as denial of service (DoS) attacks, degrade the quality of a service while causing privacy leaks [24]. When systems and devices are offline, adversaries can access sensitive information or infer more information by combining with other types of attacks. These attacks may also target the cloud endpoints and the home broadband router [25].
7. Man-in-the-middle (MITM) attack: These attacks occur when a threat agent first uses eavesdropping to learn about the communication keys used by two network peers and then impersonates one part to the other by manipulating messages and their flow, controlling the conducted communications [26]. Recently, Bettayeb et al. [27] demonstrated how certain smart sockets are prone to MITM attacks by having all the communication to/from the socket being transmitted in plaintext.
8. Eavesdropping: These attacks occur typically when the communication channels (e.g., wireless networks, Internet) are monitored to extract the content and information of a conversation. Eavesdropping may also occur through side-channel information emitted by IoT devices, e.g., power consumption and timing information. For instance, recently, researchers demonstrated a novel long-distance side-channel attacking technique called “lamphone” that can be applied to recover full sound from a victim’s room that contains an overhead hanging bulb [28].
9. Location tracing: These attacks occur when the user location is obtained or inferred, for example, by GPS spoofing or distance-attacks [29]. This can result in tracking the user possibly from anywhere there is an Internet connection, and potentially indicating the right opportunity for a break-in. Recently, the cybersecurity authority “SingCert” observed the increasing use of counterfeit contact tracing applications that can be used to steal personal user data and possibly gain knowledge of user’s whereabouts [30]. Some devices, e.g., vacuum cleaners, were also reported to produce detailed maps of the home structure and sharing these with third-parties [31].
10. Inference attack: These attacks occur when information about an ongoing task or application running on an IoT device is inferred [29]. For example, a sleep monitor activity may be scanned to reveal whether a user is awake or not [32]. This attack category may be generalized to infer other user activities, e.g., sleeping, showering, and cooking. Inference attacks can typically be performed after eavesdropping, i.e., commonly after first identifying the particular device type.

User layer attack. Residents may be the direct target of privacy attacks. Typically, this happens by having them lured to install phony applications or by having them agree to policies and terms and conditions without being aware of privacy implications. Nonetheless, these attacks may be also initiated at the smart home remote’s backend.

11. Data poisoning: In poisoning attacks, the malicious threat agents seeks to damage the integrity and confidentiality of a system, including its machine learning model [33]. Similar to device tampering, but at a higher architecture level, this attack may result in revealing sensitive data. Data poisoning attacks may also be caused by application layer attacks such as cross-site scripting and SQL injection.
12. Model inversion: By interacting with a machine learning model, subtly changing its activity, and using a technique known as model inversion, a threat agent may be able to deduce key features of the underlying data on which the system was trained, essentially gaining access to classified data [34].
13. Membership inference: This attack allows a threat agent to deduce whether a given individual is present in the training data; and not necessarily learn additional personal data; of a machine learning model [35]. This can be used for instance to detect if a particular resident belongs to a certain demographic or a certain cluster of consumers allowing for more targeted attacks.
14. Social engineering: This may result in users installing malware, e.g., in the form of fake applications or browser plugins on their devices, resulting in unauthorized access to their data. Shoulder surfing is a type of social engineering attack where sensitive data may be revealed to an intruder example by hearing sensitive information being spoken. For instance, certain smart speakers may require the user to speak a PIN code aloud for protecting voice purchasing [36]. However, this code may be overheard by others, e.g., by temporary visitors located at the home.
15. Data disclosure: A data controller with legal access to consumer data may disseminate the information legally, e.g., to advertisement firms for marketing purposes, and thus violate the privacy rights of an individual [37]. Moreover, a third-party may access the residents’ data illegally, e.g., by storing and accessing information without being given the explicit consent of the data subject [37]. For example, a recent study [38] concluded that all surveyed consumer devices expose information to eavesdroppers via at least one plaintext.

The developed taxonomy is a first approach towards categorizing privacy attacks targeting smart homes. This taxonomy is used as a core component in Section 5 to determine susceptibility to privacy attacks. In the next section, we discuss current work in relation to threats and risks associated with smart homes.

## 4. Related Work

Over the last few decades, ample research has been conducted on various aspects of privacy. From a risk management perspective, this work includes the identification and organization of privacy threats, mitigation strategies, and methods to evaluate the risk of privacy violations. This work lies at the intersection of the research areas of privacy and security threat modeling and risk analysis. Accordingly, we present an overview of the related work according to these two research areas and discuss similarities with and differences from our work.

### 4.1. Threat Modeling Models and Methodologies

Threat modeling is a process for discovering, classifying, and evaluating the risk of threats to a system from an attacker’s perspective. Originally, threat modeling was exclusively used for security purposes, however, privacy researchers have extended it to address privacy concerns [39].

The STRIDE model was proposed by Microsoft [40] as a security threat identification process classifying threats into six categories (Spoofing, Tampering, Repudiation, Information disclosure, Denial of service, and Elevation of privilege). While STRIDE is useful for performing threat analysis it is designed for security analysis and therefore its use for privacy analysis is limited.

LINDDUN [41] is a privacy threat modeling technique, which is analogous to the STRIDE model, helping in the systematic identification of privacy threats and the selection of privacy-enhancing technologies to mitigate the associated risk. The “LINDDUN” acronym is derived from the categories of privacy threats it identifies, namely: Linkability, Identifiability, Non-repudiation, Detectability, Disclosure of information, Unawareness, and Non-compliance. While LINDDUN is useful for modeling of software-based systems, it does not provide the means to evaluate risks or to analyze risks in a quantitative manner.

The Quantitative Threat Modeling Methodology (QTMM) [42] is a quantitative threat modeling methodology to objectively draw conclusions about privacy-related attacks. Similar to LINDDUN, QTMM is based on the STRIDE approach and follows the same modeling steps. Nonetheless, QTMM focuses on three privacy-specific threat categories: linkability, unawareness, and intervenability, which is restricted compared to LINDDUN. QTMM uses quantifiable attack trees, for the purpose of helping an analyst to take objective decisions about the threats, attacks, and mitigation mechanisms [42]. The use of risk-based quantification through attack trees is lacking in LINDDUN. While QTMM provides a methodology for analysing and evaluating privacy threats it does not deal with smart home specific threats.

EPIC [43] is an operational methodology that is designed to identify and evaluate privacy violation threats resulting from the deployment of an organizational cybersecurity system (CSS)–CSSs tend to handle large amounts of sensitive information dealing with the whole organization’s network traffic [43]. Specifically, EPIC is aimed at guiding security and privacy professionals with instructions from modeling data exposure in a CSS to the evaluation of privacy violation risks. Different to LINDDUN and QTMM, EPIC considers any data disclosure that can reveal sensitive information about a respondent a threat. Nonetheless, EPIC while having having similar objectives to ours, i.e., the evaluation of privacy risks, it is does not capture the dynamics of smart homes and the modeling of threat agents.

Other notable privacy threat modeling approaches and frameworks, e.g., FPFSD [44] and PriS [45] exist in scholarly literature, however, these have different goals to ours. For instance, having a focus on the organisational goals and business processes of a system (e.g., PriS [45]), and giving advise for privacy enhancing mechanisms for software architectures (e.g., FPFSD [45]). In our case, our main focus is on risk analysis. Moreover, there are other assessment based frameworks, such as SDM [46] and CNIL [47], that function as privacy impact assessment (PIA) methodologies. Specifically, SDM and CNIL are designed to build and demonstrate compliance with the EU General Data Protection Regulation (GDPR) principles [48]. Achieving compliance to the GDPR or other regulations is not the main scope of our work. Nonetheless, when we formally model the smart home in Section 5.1, we use aspects derived from the GDPR, e.g., the data processing purpose, for capturing privacy-related attributes.

Comparison to related work. In this paper, we adopt a similar approach to QTMM for modeling attacks and quantifying privacy risks. However, unlike QTMM, we model the system using a formal system specification rather than using a Data Flow Diagram (DFD). While DFDs can be used to identify threats, their use is restricted, especially when it comes to representing specific system properties that could help an automated analysis of risks. A second difference is that, unlike the previously described research works, PRASH is not intended to provide a generic or complete methodology. It is instead focused on smart homes, and it is designed to help persons who are concerned about privacy risks in their homes as a result of smart home technology implementation. Consequently, we create metrics and associated guidelines designed for measuring the privacy exposure of a smart home in a quantitative manner. For developing these metrics, and given the similar data-intensive nature of CSSs to smart homes, we use EPIC as a reference for constructing the metrics. However, different to both EPIC and QTMM, we also incorporate the dynamic element of a threat agent. An advantage of including threat agents into threat modeling is that more effective protection strategies can be identified that cater for more realistic attack scenarios.

### 4.2. Smart Home and IoT-Based Risk Analysis Models and Frameworks

Several papers focus on providing models or theoretical frameworks for the identification and analysis of risks, i.e., risk analysis, in relation to the smart home. Typically, a framework tends to give an organized structure of concepts and other things and sometimes offering guidance and direction, whereas a model represents or explains the operation and mechanism of some concept, typically developed within a framework [49]. In the majority of the reviewed literature, the development of a framework is the main contribution when it comes to risk analysis applied to smart homes (Table 2).

Denning et al. [50] provide a framework that outlines a set of emergent threats and discuss the structure of some attacks targeting smart homes arising due to the rapid introduction of connected devices. They use a scenario-driven and device-centered approach to estimate the risk. Risk in their model is calculated considering: the feasibility of conducting an attack, the attractiveness of the device as a compromised platform, and the potential damage caused by executing an attack. The first two factors when combined indicate the likelihood that an adversary will compromise a target device, and the third factor expresses the impact. This study also surveys potential computer security attacks against in-home technologies. The technological attacks identified include: direct compromise, eavesdropping, man-in-the-middle, and social engineering. While this framework provides a strategy for thinking about home security, it has different goals compared to our work. Moreover, its risk evaluation parameters, e.g., device attractiveness, tend to be subjective by nature.

Kirkham et al. [51] explore cloud computing in the context of home resource management and propose a risk-based approach to data sharing between the home and its external services. The authors designed an architecture and evaluate risk models to assist in this management of devices from a security, privacy, and resource management perspective. The proposed risk model is based on a use case for home resource management and provides means to calculate the legal risk, the appliance failure risk, and the resource security risk. While this framework architecture and risks models have been evaluated, it is use case specific making it difficult to port or extend to other smart home systems. Moreover, this study does not consider privacy attacks, and instead it focuses on resource security risks; this is represented as a summation of all probabilities of threats to the leakage of resources.

Jacobsson et al. [52] apply risk analysis on a smart home automation system. This study pointed out that human-related risks (e.g., poor password selection) and software component risks (e.g., unauthorized modification of functions in a mobile app) pose the highest risk. One conclusion of this study is that risks derived from the human factor are the most serious ones, and need more careful consideration, as they are inherently complex to handle. The analysis is conducted using the Information Security Risk Analysis (ISRA) method. While this work provides useful insights on the smart home, it is mostly concentrated on the application of risk analysis, and not on the development of a generic framework. This study, while it outlines various threats to the different smart home components, it also identifies some attacks that potentially target a smart home, namely, DoS attacks, social engineering attacks, replay attacks, and man-in-the-middle attacks.

Nurse et al. [53] outline a framework for modeling security and privacy risks in the smart home. The framework alongside its supporting prototype interface is designed to engage with smart home users and provide them with some insight into risks introduced by smart home technology devices. This could potentially result in more proactive security behavior by those users. During the threat and attack analysis phase of the framework, there is support for the inclusion of the following attacks: device tampering, information disclosure, privacy breach, DoS, identity spoofing, elevation of privilege, signal injection, and side-channel attacks. While this framework helps understanding risks and attacks, it does not model vulnerabilities in different devices, making the analysis and application to broader smart home contexts arguably limited.

Psychoula et al. [54] present a privacy modeling computation and management framework for assisted living within smart homes. The authors analyze the privacy features in the smart home that affect the privacy of the users. Based on these features, a metric is developed to compute a sensitivity score of the collected information. This metric is used in their framework to indicate risks involved in sharing the collected information and for recommending privacy settings to manage those risks. While the framework helps raise awareness and alert users about the sensitivity of information in their smart home, it does not model the feasibility of privacy attacks, e.g., in terms of their likelihood of success, and it does not consider any specific privacy attack.

Sturgess et al. [55] present a model for assessing privacy risks of a smart home based on its data-collecting capabilities (e.g., microphone, camera, and presence sensors). Then, privacy risk is given a severity score (high, medium, low, not applicable) depending on the type of personal information that is being collected and the corresponding capabilities being used for its collection. To assess privacy risk, the authors assume that any personal information exposed to the smart home is available to any adversary. Consequently, the model does not use or refer to any privacy attack. This is an assumption that simplifies the risk analysis process. However, in practice, it is difficult to accurately assign a score that correlates a capability to a set of private information, especially when excluding potential attack types. For instance, some capabilities, e.g., microphone, may be used to collect other information items than those claimed, e.g., passwords, through an eavesdropping attack.

Park et al. [29] propose a framework to measure the risk of IoT devices based on security scenarios that occur in a smart home. The authors suggest a risk measurement method and risk grade classification through the Factor Analysis of Information Risk (FAIR) method and clustering method based on the scenario. The results of this study measure the risk of possible scenarios based on security threats and assets that can be identified in the IoT-based smart home environment. This study considers four distinct attacks that can target a smart home–keystroke inference attack, task inference attack, location inference attack, and eavesdropping. While the framework contributes in measuring risk of IoT devices, its reliance on the identification of assets, threats, and misuse scenarios may limit the application of the framework to more generic instances of smart homes.

IoT frameworks. While the frameworks and models mentioned above are focused on the smart home, there are other frameworks having a broader scope but also have relevance and applicability to the smart home. Moshin et al. [56] present a framework that formally and quantitatively analyses IoT risks using probabilistic model checking. Ge et al. [57] present a framework for graphically modeling and assessing security for the IoT through formal system definitions. Recently, in 2020, targeting industry stakeholders, the National Institute of Standards and Technology (NIST) released a generic privacy framework [58] intended to help organizations manage privacy risks including those of IoT applications. Other generic research works focusing on addressing risk assessment in the IoT environment are mentioned in Kandasamy et al. [59].

Comparison to related work. In this paper, we organize the risk analysis framework and select privacy metrics in the same way that Ge et al. [57] did. However, we focus on privacy, which is an aspect that is not in that framework, and we focus specifically on the smart home as the system to model. While we consider some of the mentioned works on smart homes, e.g., Jacobsson et al. [52], our goals are to automate the risk analysis process. Different to the mentioned research works, PRASH automates the risk analysis process from the start by using attack trees that are automatically created from the smart home formal specification. It also relies on established risk modeling foundations while providing quantitative criteria for attributing the impact and attack success probability of smart home vulnerabilities. Accordingly, PRASH also leverages a risk assessment model known as DREAD [60] for measuring the privacy risk. DREAD is an acronym standing for Damage, Reproducibility, Exploitability, Affected users, and Discoverability. This method is also incorporated in some of the mentioned threat modeling methodologies [42,43,61].

Additionally, given the relationship between privacy and security, we also address requirements of security (e.g., that of confidentiality) in PRASH. To the best of our knowledge, PRASH is the first framework that uses a formal graphical model for modeling and analyzing privacy risks in smart homes, and taking into account the dynamic concept of a threat agent. Moreover, while the number of attacks featured in other frameworks tends to be rather limited (e.g., to 4 attacks in Park et al. [29] and to 8 attacks in Nurse et al. [53]) and is typically theoretical (e.g., Denning et al. [50] structure of home attacks), PRASH identifies and exhibits 15 different attack types. Another main difference between PRASH and the mentioned related work, is that PRASH uses both the CI theory of privacy and GDPR concepts to formulate its conceptual and theoretical underpinnings. This places this framework among the first to employ those foundations to determine the privacy risk exposure of a smart home. We illustrate the main differences between our framework and the other related works in Table 2.

## 5. Framework Design

The proposed framework is composed of three modules: system model, threat model, and a set of privacy metrics. The system model (Section 5.1) specifies and describes the different components of a smart home setup and their interactions. The threat model (Section 5.2) allows for the identification of privacy risks and attack paths using the system model. The privacy metrics (Section 5.3) are a set of functions that help in the evaluation of privacy risks.

While the modules are intended for people having some privacy expertise, in a real-setting, the privacy metrics may also be introduced to smart home residents, e.g., through an application with a simple graphical user interface, so that they can be involved in the decision-making process to adjust the risk parameters. Similar involvement is adopted by other risk frameworks, e.g., Nurse et al. [53] who assume a technical but non-expert smart home user is kept in-the-loop for supporting risk modeling in the smart home context.

### 5.1. System Model

We define a smart home, *S*, as a 6-tuple (H,N,U,L,D,P) where:

- H,House, represents the physical space which the residents inhabit. Formally, $H=\{z_{1},z_{2},\ldots ,z_{n}\}$, where $z_{i}\in LC$, and $z_{i}$ represents a zone, e.g., a room or a specific space, located within the curtilage of the house, where activities of daily living (cooking, eating, showering, etc.) are performed. Here, we assume the existence of a set of unique locations, LC, where the nodes in *N* can reside.
- N,Nodes, is a set of physical components that enable the smart home. Effectively, N=C∪M∪B, where *C*: connected devices, *M*: mobile devices, and *B*: backends. For *C*, this represents network-connected devices such as wireless cameras and home appliances such as Internet-enabled washing machines. For *M*, this typically represents smartphones (e.g., iPhone or Android) which are used for remotely controlling and managing *C*. For *B*, this is typically a cloud data center, but it can also be an edge device such as a dedicated home server. While *C* are located inside *H*, both *B* and *M* can be located outside *H*. Here, we assume that there are a finite set CP of capabilities, a binary relation I⊆N×CP, where I(n,cp) means that node *n* implements capability cp, and a mapping function $f_{nl}:N\to LC$. The CP for *C* were summarized earlier in Table 1.
- U,Users, is a set of human entities interacting with the smart home. A user may interact directly with *N* or indirectly through services (e.g., applications) which are incorporated in *N*. Here, we assume that there are a set *R* of roles {datasubject,datacontroller,datauser}, and a ternary relation $A_{t}\subseteq U\times R\times N$, where $A_{t}\left(u,r,n\right)$ means that user *u* has a role *r* with respect to node *n*.
- L,Links, is a set of communication channels, i.e., physical or logical channels, interconnecting *N* and *U*, and over which *D* are transferred. Effectively, L⊆(N×N)∪(N×U)∪(U×N) representing the set of data flows. Here, we assume that channels are unidirectional.
- D,Data, is a set of data items being collected and processed by *N*. Here, we assume that *D* is represented as a set of tuples $\left(d_{i},d_{s},d_{p},d_{t},d_{l},d_{e}\right)$, with the values of each attribute, except for $d_{i}$, being metadata, and where:
  – $d_{i}$, *data item*, representing the specific attributes that *N* is collects or processes. This ranges from a specific data item, e.g., name, to more generic data types, e.g., biometric data, depending on *N* [62]. An overview of data types collected by *C* is displayed in Table 3.
  – $d_{s}$, *data subject*, representing the entity, whose data are being collected or processed. This can have possible values ∈{user,system} with: user indicating that the entity represents *U* and system indicating that the entity represents *N*.
  – $d_{p}$, *data processing purpose*, representing the purpose, e.g., for uniquely identifying a person, for collecting or processing $d_{i}$.
  – $d_{t}$, *data retention time*, representing the general condition for storing $d_{i}$ with possible values ∈{indefinite,purpose,date} with indefinite indicating there is no time constraint for the deletion of the data; purpose indicating that the data has to be deleted after the completion of the corresponding purpose, i.e., after $d_{p}$ is attained; and date indicating the actual date/time for the deletion of $d_{i}$.
  – $d_{l}$, *explicit identifier*, is a Boolean indicating whether $d_{i}$ explicitly identifies the identity (social security number, voice, MAC address, etc.) of $d_{s}$.
  – $d_{e}$, *data control*, representing a set of tuples (control,phase) where control represents a privacy-enhancing technology with possible values ∈{anonymization,de−identification,encryption}, and phase indicates the data lifecycle phase, with possible values ∈{generation,collection,processing,disclosure}, over which the control is implemented.

- P,Policy, is a set of rules describing the smart home configuration and operation. Based on the trigger-action programming paradigm, and similarly to the IFTTT pattern extension proposed by Nacci et al. [63], we represent a rule as the pair–trigger and action. Trigger represents the condition, such as location, time, arrival of a specific person, or a particular property of *S*, e.g., $d{}_{p}$, for the rule to be activated. Action represents updates or invocation on the data done as a consequence of receiving a corresponding trigger. Rules can be formalized using Extended Backus–Naur form [64] as follows:
  Rule = “(“ Trigger Action ”)”.
  Trigger = AtomicFormula | “(“ BinaryLogicalOp Trigger Trigger ”)”.
  AtomicFormula = “(“ BinaryTermOp Parameter ”)”.
  Action = “(“ Read | Write | Relay ”)”.
  Read = “read” Node ParameterName [Link] | User ParameterName [Link].
  Write = “write” Node Parameter [Link].
  Relay = “relay” Parameter Node [Link].
  BinaryLogicalOp = “AND” | “OR”.
  BinaryTermOp = “=” | “!=” | “>” | “>=” | “<” | “=<”.
  Parameter = ParameterName ParameterValue.
  ParameterName = “:” String.
  ParameterValue = “?” String.

In the grammar above, we define three actions–read, write, and relay, that transmit data between *U* and *N*. The action read extracts or queries a parameter from *U* or *N*. The action write stores a parameter inside *N*. The action relay forwards a parameter to a destination *N*. Each specified action takes an optional parameter, an instance of *L*, indicating the communication channel over which a parameter is read or sent to. In practice, the value of a parameter, represented as ParameterValue in the formal grammar, could represent concrete instances of data such as media data (e.g., video), numeral data (e.g., timestamps), and binary states (e.g., online/offline).

**Figure 3:**
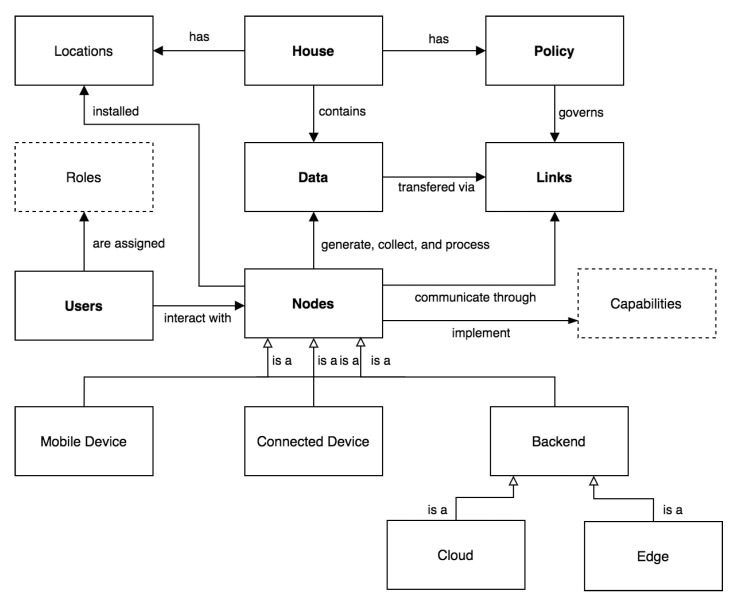
A schematic illustration of the smart home model’s components, including the logical relationships between them. Items indicated in bold represent the main attributes of the system model. Dotted boxes indicate abstract concepts.

In Figure 3, we graphically illustrate the smart home system model formalization. Effectively, the attributes of *S* can be mapped to the architecture layers described in Section 2 as follows: *N*→ hardware layer, *L*→ network layer, and *U*→ user layer. Moreover, as discussed in Section 2, we assume that *D* is pervasive across the different architecture layers. As shown in Section 6, we encoded the system model using Alloy [65], a declarative formal specification language.

**Definition 1** (*Data flow*)**.** *We define a data flow as $d_{i.r}$ where i specifies the data sender or subject and r the data recipient, and i∈(U∪N) and r∈N. Each data flow carries data items $\{d_{1},\ldots ,d_{n}\}\subseteq D$. Technically, how data flows and are interrelated in terms of data transformations are specified in P.*

**Definition 2** (*Data context*)**.** *We define a data context as $D_{c}$, consisting of a non-empty set of data flows. Effectively, $D_{c}$ represents the permissible data flows for use in a particular setting. We assume that S has a set of valid contexts, $S_{c}$ = $\left\{D_{c_{1}},\ldots ,D_{c_{n}}\right\}$ specified by the homeowner.*

For privacy risk analysis, we assume that the data contexts have been identified and labeled according to their type and sensitivity. This would allow for instance differentiating between sharing health data from a smartwatch to a doctor (e.g., represented as $D_{doctor}=\left\{d_{watch.health\_cloud}\right\}$) versus sharing health data with another entity, e.g., to the smart meter provider. The latter data context may be considered inappropriate by the data subject, whereas the former data context is appropriate. Identifying the data contexts is core to the CI theory of privacy.

### 5.2. Threat Model

Given *S*, we can identify the set of possible privacy threats, *T*, that can result in a privacy violation. This can be done for instance by having *S* specified in a property specification language and then analyzed through a model checker [66].

We assume that there is a global set of vulnerabilities, *V* for *T*. An attack exploits a set of vulnerabilities and when it is successful it results in the creation of a corresponding threat(s). Attacks targeting the smart home were established earlier and grouped in a taxonomy in Section 3. In relation to the taxonomy, we assume the existence of a function, *query-taxonomy(c,al)*, that returns a set of attack types (e.g., service attack) from the taxonomy given *c* representing the entity threatened (e.g., the network) and al representing the access level (e.g., active). We assume that al is an optional parameter.

Each $v_{i}\in V$ has a set of exploitability-relevant parameters, α, where $\alpha =\{\alpha_{l},\alpha_{i}\}$ indicating the attack success likelihood ($\alpha_{l}$) and attack impact ($\alpha_{i}$), respectively. These are calculated through the privacy metrics described in Section 5.3. In practice, data regarding these scores can be obtained from risk assessment studies, and using open repositories of vulnerabilities such as the Vulnerability Scoring System (CVSS) [67] or the National Vulnerability Database (NVD) [68].

**Definition 3** (*Attack tree*)**.** *Using the attack tree formalization proposed by Ge et al. [57] we define an attack tree, at, as a 5-tuple at=(X,Y,c,g,root). Here, X is a set of components which are the leaves of at and Y is a set of gates which are the inner nodes of at. We require X∩Y=∅ and root∈X∪Y. Let ℘(Z) denote the power set of Z. The function c:Y→℘(X∪Y) describes the children of each inner node in at (we assume there are no cycles). The function g:Y→{AND,OR} describes the type of each gate.*

In Algorithm 1, we document how the threat model is created from *S*. The algorithm assumes the existence of a helper function, *build-attack-tree(attack-goal, root, attack-types)*, that generates an at using *attack-goal* as the goal of the attack, root acting as a unique name for the at root node, and *attack-types* representing children nodes; and a function $join(at,at_{s})$ that appends a subtree $at_{s}$ to at and thus expanding at with new attack paths. Finally, we assume that the vulnerabilities of *N*, *U*, and *L* are combined using logical AND and OR gates; and that the leaf nodes of at are the identified vulnerabilities. In Algorithm 1, line 7 and 8 refer to the computation of the attack success likelihood and attack impact, respectively. While the attack impact can be calculated automatically through DecisionSupportSystem, as shown in line 8, it can be adjusted considering the parameters indicated in line 10. Details about these metrics are discussed in Section 5.3.
**Algorithm 1** Computation of attack metrics for each vulnerability.  **Input:**
*S*:smart home, $S_{c}$:data context set, ag:attack goal, al:access level
  **Output:**
at:attack tree
1:  at.root←ag2: 
$\mathrm{for each}\;\Psi_{i}\in (N\;\cup \;U\;\cup \;L$) **in**
*S*
**do**3:
  
$attack\_type.\Psi_{i}\leftarrow \mathrm{query-taxonomy}($Ψi,al)4:
  
$subtree\_at.\Psi_{i}\leftarrow \mathrm{build-attack-tree}(\mathrm{ag},$Ψi,attack_type.Ψi)5:
  
$\mathrm{if}\;subtree\_at.\Psi_{i}$ not emptythen6:
   
$\mathrm{for}\mathrm{each}v_{i}\in subtree\_at.\Psi_{i}\;\mathrm{do}$7:    
$v_{i}._{\alpha_{l}}\leftarrow$
*p(discoverability)* × *p(reproducability)* × *p(exploitability)*8:    
$v_{i}._{\alpha_{l}}\leftarrow$
*DecisionSupportSystem(P,D,S_C_)*9:    **if**
*user-override*
**then**10:     
$v_{i}._{\alpha_{l}}\leftarrow$
*Norm(affected users* × *damage potential)*11:    **end if**12:
   **end for**13:   
*join(at, subtree_at.*Ψ_i_)
14:  **end if**15:  **return**
*at*
16: **end if**

Threat agent. A threat agent, ta, is a person (or a group of persons) who originates attacks to achieve a goal related to the system under attack [69]. Each ta owns different skills and capabilities to achieve its objectives. Nonetheless, this is done by exploiting vulnerabilities in *S* typically as a result of conducting attacks as identified in Section 3. These vulnerabilities would allow ta access to data being transmitted along *L*, reading data directly from *N* when stored or being processed, and obtaining it data directly from *U*. Hereunder, we identify instances of malicious external threat agents targeting the smart home ordered according to their respective offensive capabilities [70,71,72,73].

- *Hackers.* Malicious individuals, script kiddies, and employees of an organization who may be disgruntled, nosy, or whistle-blowers. This agent is typically moved by curiosity to experiment and try things out. An example of an attack conducted by a hacker is a social engineering attack (Section 3, item 14).
- *Thieves.* Individuals that are associated with stealing mostly for personal financial gain. This agent type is typically moved by a monetary gain, acquisition of knowledge, peer recognition, and related. An example of an attack conducted by a thieve is node theft (Section 3, item 2).
- *Hacktivists.* Individuals that mainly pursue a political or social agenda often related to human rights and freedom of information. Typically, hacktivists target specific organizations or industries. An example of an attack conducted by a hacktivist is a service attack (Section 3, item 6).
- *Competitors and organized crime.* Private criminal organizations and commercial competitors (industrial spies) that compete for revenues or resources (e.g., acquisitions). Competitors and organized crime are most likely moved by financial gains and in part by terrorism motives. An example of an attack conducted by competitors and organized crime is a routing attack (Section 3, item 5).
- *Nation states.* Enemy state attackers are groups of highly sophisticated individuals that are well-funded by governments and associated with a military unit. Nation states may target the home of individuals as part of digital surveillance programs and cyberespionage campaigns. An example of an attack conducted by a nation state is eavesdropping (Section 3, item 8).

Using Rocchetto et al. [74] work on the formal extensions to the Dolev-Yao attacker model for cyber-physical systems, we can represent the actions and rules followed by, ta, and thus also the connection between ta and *S* as:

$$\frac{\mathrm{ta}(\mathrm{attacker}-\mathrm{property})\;\;S(\mathrm{system}-\mathrm{property})}{\mathrm{action}-\mathrm{result}}action$$

where: *attacker-property* represents a property of ta, e.g., access type (in-person, remote, in-network) with respect to *S*; *system-property* represents a physical or logical property of *S*, e.g., connected device status (on, off); action is the action to be performed on *S*, e.g., reading data from *N*; and *action-result* is the result of the action performed (e.g., user’s data obtained). The *attacker-property* and *system-property* act as preconditions to perform action. Properties can also be further combined using Horn clauses [74]. In practice, the success of executing an action depends on the *threat agent’s power*.

Threat agent power. Threat agents can exploit vulnerabilities in *S* and perform different actions. We assume the threat agent power, $ta_{p}$, to be a value, [0,1]. This value represents the agent’s overall familiarity with *S* and its offensive capabilities. A high value of $ta_{p}$, e.g., $ta_{p}\geq 0.7$, is indicative of ta possessing advanced knowledge of *S*, e.g., in terms of the devices used, home network configuration, and residence routines, and having advanced offensive capabilities, e.g., in terms of tools (hardware and software) and skills (e.g., technical expertise in exploiting protocols, hardware, and security services), whereas a low value of $ta_{p}$, e.g., $ta_{p}\leq 0.3$, indicates the contrary. In practice, $ta_{p}$, can also factor in other attributes, for example, the available time and monetary resources of ta for performing an attack. While we assume an aggregate value for $ta_{p}$, different attack attributes can be combined, for instance using multi-attribute utility theory [75]. Nonetheless, a high $ta_{p}$ can be associated with nation states and competitors and organized crime, and in general a low $ta_{p}$ to hackers, thieves, and hacktivists.

### 5.3. Privacy Metrics

The privacy metrics help us measure the privacy risk, i.e., the risk to the data subject after an attack is performed on *S*. Accordingly, we develop three metrics–attack success likelihood, attack impact, and risk score, described as follows.

Attack success likelihood. This metric determines the probability of ta to successfully compromise a target to achieve an attack goal and thus obtain access to the data of a data subject. We assume the attack success likelihood, $\alpha_{l}$, to be a value, [0,1]. Here, a low value of $\alpha_{l}$, e.g., $\alpha_{l}\leq 0.3$, is indicative that it is difficult and unlikely (rare) for ta to access a component whereas a high value, e.g., $\alpha_{l}\geq 0.7$, indicates the contrary, i.e., it is likely.

In order to populate $\alpha_{l}$ for the entire attack tree, we can use the aggregation rule defined by Equation (1).

(1) $$\alpha_{l}=\left\{\begin{array}{cc} \prod_{i=1}^{n}\alpha_{l.i}, & \mathrm{if}\;AND\;\mathrm{node}\\ max(\alpha_{l.i}),i=1\ldots n, & \mathrm{if}\;OR\;\mathrm{node} \end{array}\right.$$

Based on the DREAD risk assessment model [60], we adopt the risk categories of discoverability (e.g., determining how likely are the attackers to discover the vulnerability), exploitability (e.g., determining how much work is needed to implement the attack), and reproducibility (e.g., determining how reliable is the attack), for calculating $\alpha_{l}$ of a privacy attack. These parameters are combined as conditional probabilities in Algorithm 1. Guidelines for determining the probabilistic score for each parameter are found in Table 4.

Some of the capabilities of *N* can also affect $\alpha_{l}$. For instance, if *N* implements *remote access* it is more likely that this allows a ta easier access to *N*. Similarly, the more capabilities *N* supports, the more likely it is that *N* is exposed to more vulnerabilities. For instance, if *N* implements *API, IFTTT, web browser accessibility, and smartphone accessibility*, this is likely to increase the discoverability of potential attacks as the attack surface of *S* is widened.

It is also possible to positively correlate $\alpha_{l}$ to $ta_{p}$. Thus, we can evolve $\alpha_{l}$ into $\alpha_{l._{\mathrm{ta}}}$ to factor in $ta_{p}$. In this way, $\alpha_{l._{\mathrm{ta}}}$ becomes jointly dependent on *S* and ta properties. Through $\alpha_{l._{\mathrm{ta}}}$ we can more realistically compute risk scenarios based on dynamic threat agent behavior (e.g., including increasing attacker resources). Using Item Response Theory [76,77,78] we can combine the relation between $ta_{p}$ and $\alpha_{l}$ in a logistic function as follows:

(2) $$\alpha_{l._{\mathrm{ta}}}=\frac{e^{ta_{p}-\alpha_{l}}}{1+e^{ta_{p}-\alpha_{l}}}$$

Using Equation (2) we can represent scenarios where, for example, if an attack is more difficult, the $\alpha_{l}$ will be lower assuming $ta_{p}$ stays the same.

Attack impact. This metric determines the potential loss to the privacy of a data subject caused when ta manages to successfully achieve the corresponding attack goal. We assume the attack impact, $\alpha_{i}$, to be a value, [0,10]. Effectively, this equates to the maximum potential harm caused to the data subject when ta successfully compromises a target. Here, a low value, e.g., $\alpha_{i}\leq 3$, indicates that the impact is almost negligible, whereas a high value, e.g., $\alpha_{i}\geq 7$, is indicative that the impact is major.

In determining $\alpha_{i}$, we base it on the calculation of the level of identification of the data subject and data context sensitivity. Formally, we assume the existence of a decision matrix, $\gamma =M_{i_{l}d_{c}}$, for calculating the privacy impact with $i_{l}$ representing the *identification level* and $d_{c}$ representing the *data context sensitivity*. We also assume a corresponding *lookup function*, $f_{\theta }$:$i_{l},d_{c}$$\to \alpha_{i}$, for γ. While we assume that $\alpha_{i}$ is associated with a single data subject rather than the collective dimension—as is the typical case when conducting privacy risk analysis [79]—we also indicate how $f_{\theta }$ can be tuned for impact that affects a group of users, e.g., the entire family.

Identification level. The identification level, $i_{l}$, determines the extent to which a data subject can be identified from a data flow. Identification of a data subject can occur by having explicit identifiers declared in the data flow (i.e., in the corresponding $d_{l}$ attributes), or otherwise by having quasi-identifiers that can help identify a data subject indirectly with sufficient background knowledge. Some examples of possible identifiers that can used to identify a person are: email addresses, device identifiers (e.g., MAC addresses and serial numbers), biometric identifiers, and more [80]. We assume that, $i_{l}$, to be a value, [0,10]. Here, a low value indicates that explicit identity is not part of the data flow and quasi-identifying information in the data flow is unlikely to reidentify a data subject, whereas a higher value indicates the contrary and thus a possibility that reidentification of the data subject is possible. In determining whether identification data are present in a data flow, the $d_{l}$ attribute of the corresponding data items ($d{}_{i}$) of a data flow can be inspected.

Data context sensitivity. The data context sensitivity, $d_{c}$, identifies the violation to privacy as perceived by the data subject in a specific context. We assume that, $d_{c}$, to be a value, [0,10]. Concurring with the CI theory of privacy [81] we associate $d_{c}$ to the intended use of the collected data. Thus, this value equates to the whole context of the data flow, instead of the individual data element. Here, a low value, indicates that the data are being used/processed in a context that is assessed by the data subject as involving a low impact (e.g., sharing energy profile of the home with the smart grid supplier), whereas a high value indicates the contrary and thus data are being used in a critical context (e.g., sharing medical data with a healthcare provider) implying that the user privacy can be at stake depending on $i_{l}$. The data context sensitivity may also depend on the location where a node is installed (e.g., the bathroom might be considered a more sensitive context than the kitchen room). Here, we assume the existence of an oracle that can identify the current context of a data flow, and by inspecting the smart home policy (*P*) can determine whether the data flow respects the designated context or not.

Lookup function. The impact is determined by $f_{\theta }$ using Table 5 as γ. In practice, Table 5 serves as guidance and the data subject can adjust the particular weights according to their own judgement. A similar approach is followed by EPIC [43] for building the privacy likelihood matrix. However, we also include additional suggestions to further refine the output produced by $f_{\theta }$ and thus the final score for $\alpha_{i}$. Based on the DREAD model [60] we include damage potential (e.g., determining how much the attack costs to the family) and affected users (e.g., determining how many people are impacted by the attack) as potential impact factors for $\alpha_{i}$. The mentioned risk factors and $f_{\theta }$ were integrated earlier Algorithm 1. In Algorithm 1, the DecisionSupportSystem is effectively the implementation of the lookup function and decision matrix.

For damage potential, we associate this to the monetary loss, psychological, and potentially safety harms caused by an attack, and grade this using an ordinal scale (0–5). We assume that a higher value of damage potential is associated also as to when there are vulnerable data subjects involved. Some examples of vulnerable data subjects are children, employees, and people needing special protection [82]. The damage potential can also be calculated based on the data items ($d{}_{i})$ that *N* collects and processes, and the data subjects ($d{}_{s}$) corresponding to those. Leaking certain $d{}_{i}$ can cause a direct threat to user privacy, for example, by revealing patterns of social life, behaviors and actions, the state of one’s body and mind, etc. [83]. Arguably, the more of these aspects that are affected, the more likely it is for an increased damage potential. Nonetheless, if there are data controls ($d{}_{e}$) set across the different data lifecycle phases of *S* then the damage potential is likely to become lower than when not set.

For affected users, we assume that this represents whether the leaked data affects one individual to multiple users, e.g., the entire family, and grade this using an ordinal scale (1–3). The affected users is also related to the smart home backend (i.e., *B*). For instance, if *B* is a cloud backend, then it is more likely that a group (2) is affected. It can also be argued that if a node (*N*) implements *gateway functionality* as a capability then arguably more users, e.g., the entire family, could be affected in case *N* is compromised.

In Table 6, we display the risk factors associated with $\alpha_{i}$. These factors are combined using multiplication, and thus $\alpha_{i}$ = *Norm(damage potential × affected users)*, with Norm being a function that normalizes the output into the range of [1–10].

In order to populate $\alpha_{i}$ for the entire attack tree, we can use the aggregation rule defined by Equation (3).

(3) $$\alpha_{i}=max\left(\alpha_{i.i}\right),i=1\ldots n,\mathrm{for}\;\mathrm{both}\;AND\;\mathrm{or}\;OR\;\mathrm{node}$$

Risk score. Following a common approach in computer security, we compute the privacy violation risk as the combination of likelihood of occurrence of a privacy violation and its impact. Specifically, by multiplying $\alpha_{l}$ with $\alpha_{i}$, we come up with a quantitative score ($r_{\mu }$) representing the risk level of a smart home component. This value is indicative of the priorities that should be invested in making the smart home secure against the discovered vulnerabilities. Scores range from 0 to 10, with 10 being the most severe. In Table 7, we provide guidelines on how the risk scores can be described.

In order to populate $r_{\mu }$ for the entire attack tree, we can use Equation (4).

(4) $$r_{\mu }=\alpha_{l.\mu }\times \alpha_{i.\mu },\mu =1\ldots n$$

## 6. An Application of PRASH

In this section, we demonstrate the usefulness of the proposed framework for analyzing privacy risks of smart homes. We start by describing how we generated a smart home instance from the formal system model specification (Section 6.1). Next, we present the threat model (Section 6.2) for that instance. Finally, we reveal the privacy metrics and use those to summarize the risks found in the smart home instance (Section 6.3).

### 6.1. System Model

To generate a sample smart home we used the open source language and analyzer called Alloy as our formal specification language. Three alternatives to it are B, TLA+, and Z. However, we adopted Alloy in particular as it supports, through its automated tool called Alloy Analyzer, the generation of graphical results which are convenient for analysis work. Moreover, we used Alloy to capture the specifications of the smart home and the structural relations between its various components. The source code we developed and used for specifying and generating a smart home system model is available on Github (https://github.com/bugejajoseph/smarthome (accessed on 8 September 2021)).

For the smart home *S*, we considered a realistic use case consisting of a connected toy and a video doorbell as the main smart home devices. The connected toy was a voice interactive toy used by a child for personalized entertainment and learning purposes. The video doorbell was an outdoor camera that automatically notified the parent (homeowner) when a visitor arrived at the door. Live footage from the video doorbell was sent to the mobile phone of the parent. Both the connected toy and video doorbell were connected to the cloud. By using the system model described in Section 5.1, we can represent the described use case as follows:

- House = {KidRoom,FrontDoor,LivingRoom}
- Nodes = {MobileDevice,ConnectedToy,VideoDoorbell,Cloud}
- Users = {Child,Parent,ServiceProvider}
- Links = {Link0,Link1,Link2,Link3}
- Data = {
  (motion,user,detect presence of visitors,purpose,false,∅),
  (video,user,video footage of visitors,purpose,false,{(encryption,disclosure)}),
  (audio,user,conversation between child and connected toy,indefinite,true,∅),
  (control,system,smart home states and parameters,purpose,false,∅)

}

The data items represent instances of the smart home data types household data, biometric data, and other data, which we identified earlier in Table 3.

- Policy = {
  $r{}_{1}$ = (= :motion_detected ?true) read VideoDoorbell :video_footage
  $r{}_{2}$ = (!= :video_footage ?empty) relay :video_footage ?video MobileDevice
  $r{}_{3}$ = (= :listening_on ?true) relay :conversation ?audio Cloud
  $r{}_{4}$ = (!= :conversation ?empty) relay :conversation ?audio ConnectedToy

}

The policy indicates that when motion is detected by VideoDoorbell, the corresponding video footage is automatically captured ($r_{1}$) and is made available to MobileDevice ($r_{2}$). Furthermore, audio data are relayed to the Cloud ($r_{3}$), which are subsequently used to interact back with the child through the ConnectedToy ($r_{4}$).

**Figure 4:**
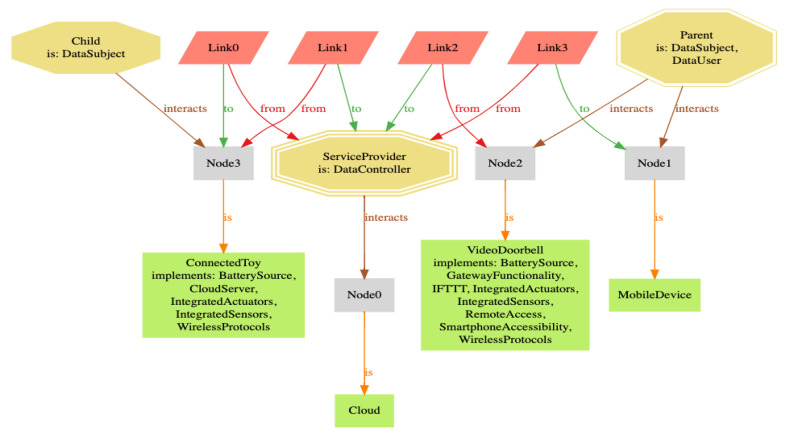
A smart home configuration generated using Alloy. The smart home setup consists of 4 nodes (ConnectedToy, VideoDoorbell, MobileDevice, Cloud), 3 users (Child, Parent, ServiceProvider), and 4 links (Link0-Link3) that interconnect users to nodes, and vice versa. All the relations between the different model components is displayed in the form of labelled arrows.

Though Alloy we encoded the described smart home setup as shown in Figure 4. For simplicity, we focused on representing nodes, links, and users as our main entities. Nonetheless, we also added capabilities, as per Table 1, to the connected devices, mapped the users to the nodes, and assigned roles to users.

We can assume $S_{c}$ = {EntertainmentCxt,SecurityCxt} where $EntertainmentCxt=\{audio_{Child.ConnectedToy},audio_{ConnectedToy.Cloud},audio_{Cloud.ConnectedToy}$} and $SecurityCxt=\{video_{VideoDoorbell.Cloud},motion_{VideoDoorbell.Cloud},video_{Cloud.MobileDevice}\}$. These contexts indicate how the different data items are exchanged in *S*.

Finally, we assume LC={KidRoom,FrontDoor,LivingRoom,ServiceProvider}. Consequently, we assume Cloud to be located in ServiceProvider, and MobileDevice,

VideoDoorbell, and ConnectedToy are located in LivingRoom, FrontDoor, and KidRoom, respectively.

### 6.2. Threat Model

**Figure 5:**
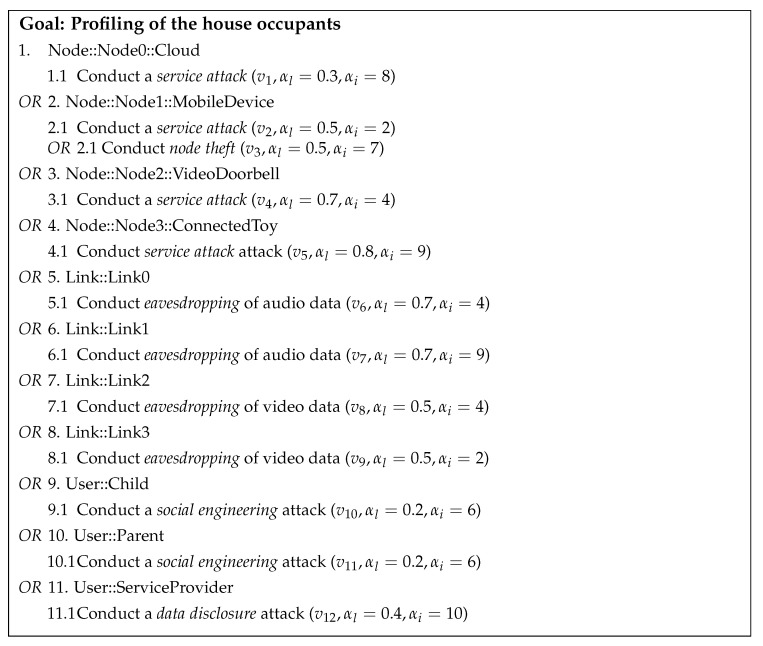
Attack tree with the attacker’s goal being that of profiling the house occupants.

In our threat model, let us assume that the attack goal is that of profiling of the house occupants. Profiling is a threat of collecting and correlating information about individual activities, and subsequently using them to generate new information from the original data [66]. Aggregated profiles of individuals can constitute a risk to a person’s privacy, particularly if children are involved. We created an attack tree for this attack (Figure 5) through Algorithm 1.

In Figure 5, we assume that the system model, *S*, is that described in the previous section. The attack tree describes how the different components of the model can be compromised to achieve the attack’s goal. We assumed that the attack is conducted by exploiting a set of hypothetical vulnerabilities ($v_{1},\ldots ,v_{12}$). For the assigned values of $\alpha_{l}$ and $\alpha_{i}$, we based these on specifications of *S* and on data derived from vulnerability databases, when possible, as explained below:

- Node capabilities, location, and users. We considered the specifications of *S*, particularly, the device’s capabilities, location, and user types. For instance, the VideoDoorbell had embedded gateway functionality, IFTTT, and supports remote access. These capabilities could add up to a higher $\alpha_{l}$ (due to the broadened attack surface). Nonetheless, $\alpha_{i}$ was low, as the location was not particularly sensitive (outdoors) and the users were visitors, not necessarily family members. The rationale for calculating $\alpha_{l}$ and $\alpha_{i}$ was detailed in Section 5.3.
- Data contexts, policy, and controls. We consider dynamic aspects captured in *S*, particularly, those related to the data, policy, and contexts. For instance, the ConnectedToy is sending audio data, which is indicated to identify a child, unencrypted, and that makes $\alpha_{i}$ high. Furthermore, the context EntertainmentCxt and policy rules $r{}_{3}$ and $r{}_{4}$ indicate that this data are sent to the cloud and back, indicating a risk that sensitive data, potentially of the entire family, are being channeled out of the home and without using any privacy related controls (e.g., anonymization). Thus, $\alpha_{l}$ is also considered high. In practice, the DecisionSupportSystem can implement some of this logic automatically.
- Vulnerability databases. We used vulnerability databases for finding actual/practical instances of weakness of smart home devices. A search for vulnerabilities for smart home devices embedding cameras revealed various instances where such cameras have been repeatedly exploited. For example, $\alpha_{l}$ of exploiting vulnerability CVE-2015-2887 (https://www.cvedetails.com/cve/CVE-2015-2887 (accessed on 8 September 2021)), targeting a certain type and brand of connected camera, was reported as critical, and thus associated with high $\alpha_{l}$. In practice, searching for vulnerabilities affecting smart home devices can be done automatically [84].

Moreover, to implement the attacks, we assumed $ta_{1}=\left\{hacker\right\}$ with $ta_{1p}=0.2$, and $ta_{2}=\left\{\mathrm{nation}-\mathrm{state}\right\}$ with $ta_{2p}=0.8$. In terms of actions and rules followed by $ta_{1}$ and $ta_{2}$, we assumed that both can learn an attribute (e.g., user credentials) of *N* or *L* without being physically present inside the house.

$$\frac{ta(distance,remote)\;\;S(attrib,stored/processed\;in\;N)}{ta\;learns\;attrib\;ofN}read\left(attrib,N\right)$$

$$\frac{ta(distance,remote)\;\;S(attrib,transmitted\;on\;L)}{ta\;learns\;attrib\;of\;L}read\left(attrib,L\right)$$

Moreover, $ta_{2}$ can learn an attribute (e.g., personal and sensitive data) of *U* by being physically located inside the home network.

$$\frac{ta(distance,\mathrm{in}-\mathrm{network})\;\;S(attrib,is\;known\;by\;U)}{ta\;learns\;attrib\;of\;U}listen\left(attrib,U\right)$$

### 6.3. Privacy Risk Analysis

Based on the privacy metrics established in Section 5.3, we could calculate the overall attack success likelihood and attack impact, and thereby describe the risks as follows:

Attack success likelihood: We calculated $\alpha_{l}$ by Equation (1). 



$\alpha_{l}$

 = $max(\alpha_{l.v_{1}},max\left(\alpha_{l.v_{2}},\alpha_{l.v_{3}}\right),\alpha_{l.v_{4}},\alpha_{l.v_{5}},\alpha_{l.v_{6}},\alpha_{l.v_{7}},\alpha_{l.v_{8}},\alpha_{l.v_{9}},\alpha_{l.v_{10}},\alpha_{l.v_{11}},\alpha_{l.v_{12}})$ = max(0.3,max(0.5,0.5),0.7,0.8,0.7,0.7,0.5,0.5,0.2,0.2,0.4) = max(0.3,0.5,0.7,0.7,0.8,0.7,0.5,0.5,0.2,0.2,0.4) = 0.8

The computational results indicate that the most likely target for achieving the attack goal with 80% success rate is by attacking the ConnectedToy through vulnerability $v_{5}$. The ConnectedToy potentially could be using insecure protocols, default passwords; it may have firmware that is not updateable; and more. Effectively, this may translate to: p(discoverability) = 1, p(exploitability) = 0.8, and p(reproducability) = 1. Since both $ta_{1}$ and $ta_{2}$ have remote access to the home, this attack is possible to conduct by both.

Next, we applied Equation (2) to calculate $\alpha_{l._{\mathrm{ta}}}$ for $ta_{1}$ and $ta_{2}$ for exploiting vulnerability $v_{5}$. The results are $\alpha_{l._{\mathrm{ta}}1}$ =$\frac{e^{0.2-0.8}}{1+e^{0.2-0.8}}$≈ 0.35; $\alpha_{l._{\mathrm{ta}}2}$ =$\frac{e^{0.8-0.8}}{1+e^{0.8-0.8}}$≈ 0.5. Consequently, this indicates that $ta_{2}$, as expected, has a better chance of achieving its goal than $ta_{1}$.

Attack impact: We calculate $\alpha_{i}$ by Equation (3). 



$\alpha_{i}$

 = $max(\alpha_{i.v_{1}},max\left(\alpha_{i.v_{2}},\alpha_{i.v_{3}}\right),\alpha_{i.v_{4}},\alpha_{i.v_{5}},\alpha_{i.v_{6}},\alpha_{i.v_{7}},\alpha_{i.v_{8}},\alpha_{i.v_{9}},\alpha_{i.v_{10}},\alpha_{i.v_{11}},\alpha_{i.v_{12}})$ = max(8,max(2,7),4,9,4,9,4,2,6,6,10) = max(8,7,4,9,4,9,4,2,6,6,10) = 10

The computational results indicate that the most severe impact to privacy is when a data disclosure attack targets the service provider. Potentially, this attack could reveal aggregated data of multiple families, including past, current, and inferred data about vulnerable data subjects and other subjects. Effectively, this may translate to: *affected users* = 2 and *damage potential* = 5. Nonetheless, conducting an attack targeting a service provider might be challenging, as they are likely to have personnel professionally trained on privacy and security, who aware of potential legal, compliance, and regulatory ramifications.

**Figure 6:**
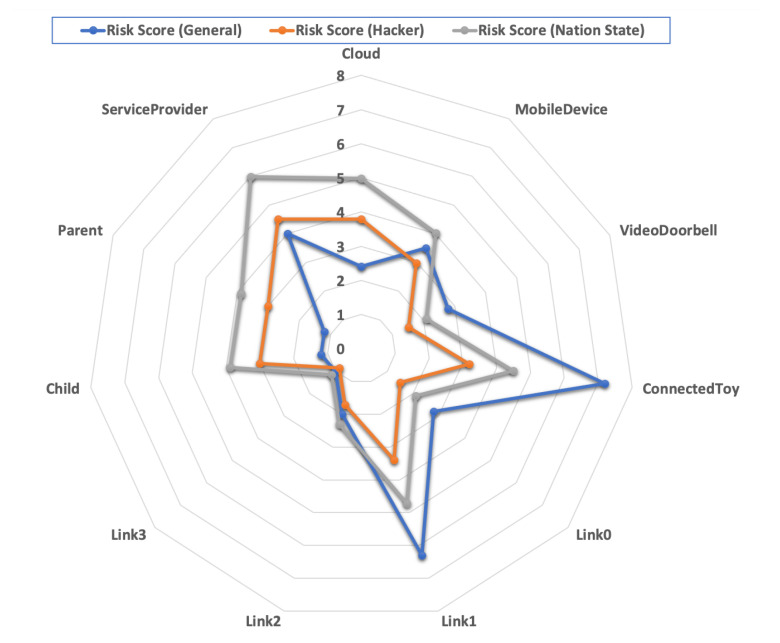
Radar chart indicating the risk level associated with each smart home component, including the risk score adjusted for the hacker and nation state actor. This figure shows that the highest risk (risk score > 7) is that of an attack targeting the ConnectedToy. Therefore, the most priority should be put on securing the ConnectedToy.

Risk scores: Using Equation (4) we computed the risk scores ($r_{\mu }$) for the entire attack tree displayed in Figure 5. The risk score for each component of the smart home is displayed in Figure 6. The results indicate that securing the ConnectedToy (followed by securing Link1) should be the top priority for making the smart home more privacy-preserving and secure. In practice, this might mean to connect the ConnectedToy to a separate segregated network, and potentially replacing Link1 with a virtual private network connection.

## 7. Extensions and Limitations

While the proposed privacy risk analysis framework is useful for better understanding the type of attacks that can target the smart home and in analyzing privacy risks therein, our research does not come without limitations.

Attack taxonomy. There are multiple ways for organizing privacy attacks. The approach we have taken is admittedly biased towards the selected dataset, primarily consisting of scholarly articles that we have analyzed. Consequently, the selected dataset may have excluded certain attack types, such as coordinated or interdependency-based attacks, that could be theoretically harnessed to invade user privacy as well. Moreover, while the taxonomy is focused on the compromise of user privacy, some attacks, as mentioned in Section 3, may also violate the security and safety of smart home residents. For instance, confidentiality, which is a main security goal, affects privacy, as the unauthorized access to some data may reveal the identity of a user. Likewise, some privacy attacks, e.g., location tracing, may compromise the safety of the occupants, for instance by having a threat agent stalk and harass victims in their homes.

System model. In the smart home system model, we did not explicitly represent the decision logic, i.e., the control algorithms, that are responsible for controlling and satisfying the system level specific constraints (e.g., safety, security, and privacy-preserving behavior). Instead, we assumed that such services, rightly so, reside in the nodes, and that their inputs/outputs consist of data. This is akin to a black box modeling approach where the focus is on the interface and the messages being exchanged, which we represented as policy rules using a formal grammar, instead of the internal behavior of the system. Nonetheless, we represent the capabilities, which may act as an enabler for an attack to occur, as attributes in the nodes. This approach allows for extending the model in the future to cater for additional threats, including concrete instances of nodes.

Threat model. Similarly to other works on quantitative risk modeling, we make use of attack trees for quantifying privacy risks. While this approach is useful, it is worthwhile considering other alternatives. For instance, attack-defense trees that include countermeasures within the attack tree. Attack-defense trees are useful for conducting risk assessment, and thus going beyond risk analysis. Moreover, we assumed a threat agent that has a global threat agent power parameter. While this works, to simulate more advanced use cases an extension of the threat model could leverage for instance, Hidden Markov Models, to represent dynamic behavior changes. Moreover, while the constructed threat model, can also cater for physical attacks, focusing on threats targeting directly the users, e.g., the threat of coercion to make a user yield certain information, is not the main scope of our work.

Privacy expertise. PRASH was primarily designed for persons who have some privacy expertise. These users tend to have experience with privacy by design and knowledge of secure development practices. Especially for some IoT vendors, in particular startups, this privacy expertise, might not be available. Nonetheless, such knowledge requirements may not be necessary for all the framework modules. Additionally, we also assume that smart home residents are the designated owners, and thus the accountable entities, of the smart home. In practice, this implies that they are also somewhat involved in the risk analysis process. However, in our case, we assume that this involvement is only needed to override certain parameters of the framework, in particular with respect to the attack impact.

## 8. Conclusions and Future Work

Smart homes can contribute to improving the quality of life of individuals. Nonetheless, smart homes challenge the notion of the home as a private and protected space. Smart homes are vulnerable to diverse privacy risks that are challenging to identify and analyze, especially given the dynamic and evolving features of their enabling IoT technologies and the processes supporting those. Accordingly, we proposed a framework called PRASH for modeling and assessing the privacy risks of smart homes. This framework uses as input a system model, a threat model, and a set of privacy metrics for helping with automating the discovery and evaluation of privacy risks affecting such systems. The capabilities of PRASH for describing a smart home for privacy risk analysis were demonstrated through a use case involving a smart home that was automatically generated through Alloy, and consequently its risks were computed. Modeling the smart home with a formal specification enables early identification of threats, better planning for risk management scenarios, and mitigation of potential impacts caused by attacks before they actually hit the homes and impact lives of residents. Overall, the proposed framework contributes to advancing the research in the area of risk analysis as applied to smart homes, and helps deepen the understanding and reasoning about privacy concerns affecting such systems.

For future work, it would be useful to develop a tool that automatically creates attack trees from a system model instantiation. This could be done by traversing the different elements of the proposed model and representing them on a graph to, e.g., illustrate the weak points of any given smart home. The computation of the different attack metrics can be done partially automatically, particularly for the nodes, by harnessing vulnerability databases such as the CVSS or NVD. A second avenue for future work would be to investigate the best ways to present risk analysis results to non-technical users and also how to communicate risks to users when they occur. Having the users engaged with the risk analysis process may contribute to increasing the trust in smart homes. Finally, it would be beneficial to evaluate the framework in a setting involving different stakeholders, e.g., smart home developers, service providers, and residents. This could serve as a means of better assessing the feasibility and usefulness of the presented framework. Potentially, in order to achieve this, prototypes could be developed and a participatory design approach could be employed.

## Figures and Tables

**Table 1:** An overview of capabilities supported by smart home devices.

Capability	Description
Gateway functionality	Provides built-in gateway functionality permitting other devices to be interconnected through it, without requiring additional hardware dependencies
Battery source	Uses a battery as its primary power source
Integrated sensors	Embeds sensors, e.g., motion detectors
Integrated actuators	Embeds actuators, e.g., electrical motors
Wireless protocols	Supports wireless protocols, e.g., Wi-Fi, to transfer information
Wired protocols	Supports wired protocols, e.g., Ethernet, to transfer information
Cloud/server	Communicates with an external backend server, e.g., for cloud storage purposes
API	Exposes a software interface that could allow for its programmatic access, control, and management
IFTTT	Provides the facility for users to create chains of conditional statements triggered by changes that occur within other web services
Web browser accessibility	Provides access via a web browser, e.g., Firefox
Smartphone accessibility	Provides access via a smartphone application, e.g., iOS application
Remote access	Supports remote access, control, and management over the Internet

**Table 2:** Contributions of PRASH in relation to the existing research work on smart homes and IoT-applicable risk analysis models and frameworks.

Research Work	Requirements	Risk Factors	Contribution	Analysis Type	Domain
Denning et al. [50]	Security	Attack feasibility, target attractiveness, and damage potential	Framework	Scenario-based	Smart home
Kirkham et al. [51]	Security and privacy	Attack feasibility, target attractiveness, and damage potential	Framework	Use cases and data-based assessments	Smart home
Jacobsson et al. [52]	Security and privacy	Based on the ISRA method	Empirical evaluation	Architecture-based	Smart home
Nurse et al. [53]	Security and privacy	Attack impact and likelihood scores	Framework	Scenario-based	Smart home
Psychoula et al. [54]	Privacy	Data item sensitivity	Framework	User-driven	Smart home
Sturgess et al. [55]	Privacy	Data-collecting capabilities	Model	Device-capabilities	Smart home
Park et al. [29]	Privacy	Based on the FAIR method	Framework	Scenario-based	Smart home
Mohsin et al. [56]	Security	Vulnerability scores, system configuration, and attacker’s capabilities	Framework	Probabilistic model checking	IoT systems
Ge et al. [57]	Security	Attack success probability, attack cost, attack impact, and mean-time-to-compromise	Framework	Formal graphical model	IoT systems
NIST [58]	Privacy	Problematic data action, attack likelihood, and attack impact	Framework	Requirements	Generic
PRASH	Privacy and security	Attack impact, attack success likelihood, and threat agent power	Framework	Formal graphical model	Smart home

**Table 3:** An overview of data types collected by smart home devices.

Data Type	Data Item Examples
Household data	Family preferences, routines, and house setup (e.g., room name)
Family data	Photos, music, and games
Biometric data	Audio, video, and health-related data
Contextual data	User location, device characteristics, and network information
Other data	Command and control data, user queries, and logs

**Table 4:** Guidelines for grading the attack success likelihood ($\alpha_{l}$).

Attack Success Likelihood	Description	Prob. Score (*p*)
Discoverability (D)	The vulnerability exists in the most commonly used feature of the smart home device, it is very noticeable, and there is published information explaining the attack	0.7–1.0
	The vulnerability is in a rarely utilized area of the smart home device, malicious usage would necessitate some thought, and only a few users should come across it	0.4–0.69
	The vulnerability is obscure, and users are unlikely to figure out the damage potential	0.0–0.39
Reproducibility (R)	The attack can be reproduced every time without requiring any privileges, timing window, or any user interaction (e.g., the rebooting the smart home device)	0.7–1.0
	The attack can be reproduced, but requires the attainment of basic user privileges, timing window, and may demand some user interaction	0.4–0.69
	The attack is very difficult to reproduce, requiring the attainment of high privileges (e.g., administrative), user interaction, and possibly requiring the threat agent to physically touch or manipulate the vulnerable smart home device	0.0–0.39
Exploitability (E)	A novice programmer could make the attack in a short time, commonly using free online resources	0.7–1.0
	A skilled programmer could make the attack, then repeat the steps	0.4–0.69
	The attack requires an extremely skilled person, special equipment, and in-depth knowledge of the smart home device and/or the home area network every time to exploit	0.0–0.39
$\alpha_{l}$	*p*(D) ×p(R) ×p(E); the score can be adjusted depending on the threat agent power ($ta_{p}$)	

**Table 5:** An example of a decision matrix for calculating the privacy attack impact ($\alpha_{i}$).

Ident. Level ($i_{l}$)		Data Context Sensitivity ($d_{c}$)			
	Negligible	Low	Medium	High	Critical
critical	1	5	7	9	9
high	1	3	5	7	9
medium	1	3	5	5	7
low	1	3	3	3	5
negligible	1	1	1	1	1

**Table 6:** Guidelines for grading the attack impact ($\alpha_{i}$).

Attack Impact	Description	Quantitative Score
Affected users (U)	The entire family and potentially other relatives and people associated with the family (e.g., friends), are affected	3
	Multiple members of the family are affected	2
	One resident is affected	1
Damage potential (D)	Personal and/or sensitive household information are leaked, including potential information about vulnerable data subjects, causing direct, serious monetary, psychological, and/or safety harms to the affected users	4–5
	Some personal or sensitive household information is leaked, however they do not include any vulnerable data subject, and the disclosure does not cause a direct or serious loss to the privacy of any resident	2–3
	Trivial information is leaked, and there is no perceived psychological or safety impact to any resident	0–1
$\alpha_{i}$	Norm(A × D)	

**Table 7:** Smart home component severity rating score ($r_{\mu }$).

Severity Score	Description
7.0–10.0	Vulnerabilities in the smart home component can be easily exploited to jeopardize user privacy
4.0–6.9	Vulnerabilities in the smart home component may be more difficult to exploit, yet they can still endanger user privacy under certain conditions
0.1–3.9	Vulnerabilities in the smart home component are believed to necessitate improbable circumstances in order to be exploited, or where a successful exploit would result in minimal consequences to user privacy
0.0	There is no risk in using the smart home component

## References

[1] Bugeja J., Jacobsson A., Davidsson P. (2018). Smart Connected Homes. Internet of Things A to Z.

[2] Zion Market Research Global Smart Home Market Worth USD 53.45 Billion by 2022. https://www.zionmarketresearch.com/news/smart-home-market.

[3] Ling Z., Luo J., Xu Y., Gao C., Wu K., Fu X. (2017). Security vulnerabilities of internet of things: A case study of the smart plug system. IEEE Internet Things J..

[4] Notra S., Siddiqi M., Gharakheili H.H., Sivaraman V., Boreli R. An experimental study of security and privacy risks with emerging household appliances. Proceedings of the 2014 IEEE Conference on Communications and Network Security.

[5] Sivaraman V., Chan D., Earl D., Boreli R. Smart-phones attacking smart-homes. Proceedings of the 9th ACM Conference on Security & Privacy in Wireless and Mobile Networks.

[6] Alrawi O., Lever C., Antonakakis M., Monrose F. Sok: Security evaluation of home-based iot deployments. Proceedings of the 2019 IEEE Symposium on Security and Privacy (SP).

[7] Nurse J.R., Creese S., De Roure D. (2017). Security risk assessment in Internet of Things systems. IT Prof..

[8] Nicklas J.P., Mamrot M., Winzer P., Lichte D., Marchlewitz S., Wolf K.D. Use case based approach for an integrated consideration of safety and security aspects for smart home applications. Proceedings of the 2016 11th System of Systems Engineering Conference (SoSE).

[9] Lopez J., Rios R., Bao F., Wang G. (2017). Evolving privacy: From sensors to the Internet of Things. Future Gener. Comput. Syst..

[10] Bugeja J., Davidsson P., Jacobsson A. Functional Classification and Quantitative Analysis of Smart Connected Home Devices. Proceedings of the 2018 Global Internet of Things Summit (GIoTS).

[11] Bugeja J., Jacobsson A., Spalazzese R., Arai K., Kapoor S., Bhatia R. (2020). On the Analysis of Semantic Denial-of-Service Attacks Affecting Smart Living Devices. Intelligent Computing.

[12] Li C., Palanisamy B. (2018). Privacy in internet of things: From principles to technologies. IEEE Internet Things J..

[13] Kenneally E. (2018). Privacy and Security. IEEE Internet Things Mag..

[14] Ziegeldorf J.H., Morchon O.G., Wehrle K. (2014). Privacy in the Internet of Things: Threats and challenges. Secur. Commun. Netw..

[15] Toch E., Bettini C., Shmueli E., Radaelli L., Lanzi A., Riboni D., Lepri B. (2018). The privacy implications of cyber security systems: A technological survey. ACM Comput. Surv. (CSUR).

[16] Nissenbaum H. (2004). Privacy as contextual integrity. Wash. L. Rev..

[17] Solove D.J. (2010). Understanding Privacy.

[18] Stallings W., Brown L., Bauer M.D., Bhattacharjee A.K. (2012). Computer Security: Principles and Practice.

[19] Arias O., Ly K., Jin Y. (2017). Security and privacy in IoT era. Smart Sensors at the IoT Frontier.

[20] Jain A., Singh T., Sharma S.K. Threats Paradigmin IoT Ecosystem. Proceedings of the 2018 7th International Conference on Reliability, Infocom Technologies and Optimization (Trends and Future Directions) (ICRITO).

[21] Mosenia A., Jha N.K. (2016). A comprehensive study of security of internet-of-things. IEEE Trans. Emerg. Top. Comput..

[22] Zhao M., Li T., Abu Alsheikh M., Tian Y., Zhao H., Torralba A., Katabi D. Through-wall human pose estimation using radio signals. Proceedings of the IEEE Conference on Computer Vision and Pattern Recognition.

[23] Arshad J., Azad M.A., Amad R., Salah K., Alazab M., Iqbal R. (2020). A Review of Performance, Energy and Privacy of Intrusion Detection Systems for IoT. Electronics.

[24] Qu Y., Yu S., Zhou W., Peng S., Wang G., Xiao K. (2018). Privacy of things: Emerging challenges and opportunities in wireless Internet of Things. IEEE Wirel. Commun..

[25] Geneiatakis D., Kounelis I., Neisse R., Nai-Fovino I., Steri G., Baldini G. Security and privacy issues for an IoT based smart home. Proceedings of the 2017 40th International Convention on Information and Communication Technology, Electronics and Microelectronics (MIPRO).

[26] Tuna G., Kogias D.G., Gungor V.C., Gezer C., Taşkın E., Ayday E. (2017). A survey on information security threats and solutions for Machine to Machine (M2M) communications. J. Parallel Distrib. Comput..

[27] Bettayeb M., Waraga O.A., Talib M.A., Nasir Q., Einea O. IoT Testbed Security: Smart Socket and Smart Thermostat. Proceedings of the 2019 IEEE Conference on Application, Information and Network Security (AINS).

[28] Nassi B., Pirutin Y., Shamir A., Elovici Y., Zadov B. (2020). Lamphone: Real-Time Passive Sound Recovery from Light Bulb Vibrations. IACR Cryptol. ePrint Arch..

[29] Park M., Oh H., Lee K. (2019). Security risk measurement for information leakage in IoT-based smart homes from a situational awareness perspective. Sensors.

[30] E Hacking News Fake Applications Are Replicating “TraceTogether,” a Singapore COVID-19 Contact Tracing Application. https://www.ehackingnews.com/2020/06/fake-applications-are-replicating.html.

[31] Maggie A. Your Roomba May Be Mapping Your Home, Collecting Data That Could Be Shared. The New York Times. https://www.nytimes.com/2017/07/25/technology/roomba-irobot-data-privacy.html.

[32] Panwar N., Sharma S., Mehrotra S., Krzywiecki Ł., Venkatasubramanian N. (2019). Smart Home Survey on Security and Privacy. arXiv.

[33] Papernot N., McDaniel P., Sinha A., Wellman M.P. SoK: Security and privacy in machine learning. Proceedings of the 2018 IEEE European Symposium on Security and Privacy (EuroS&P).

[34] Buchanan B. (2020). A National Security Research Agenda for Cybersecurity and Artificial Intelligence. Cent. Secur. Emerg. Technol. Issue Brief.

[35] Shokri R., Stronati M., Song C., Shmatikov V. Membership inference attacks against machine learning models. Proceedings of the 2017 IEEE Symposium on Security and Privacy (SP).

[36] Huang Y., Obada-Obieh B., Beznosov K. Amazon vs. My Brother: How Users of Shared Smart Speakers Perceive and Cope with Privacy Risks. Proceedings of the 2020 CHI Conference on Human Factors in Computing Systems.

[37] Asif W., Ray I.G., Rajarajan M. An attack tree based risk evaluation approach for the internet of things. Proceedings of the 8th International Conference on the Internet of Things.

[38] Ren J., Dubois D.J., Choffnes D., Mandalari A.M., Kolcun R., Haddadi H. Information exposure from consumer iot devices: A multidimensional, network-informed measurement approach. Proceedings of the Internet Measurement Conference.

[39] Hamidi F., Poneres K., Massey A., Hurst A. Who should have access to my pointing data? Privacy tradeoffs of adaptive assistive technologies. Proceedings of the 20th International ACM SIGACCESS Conference on Computers and Accessibility.

[40] Snyder W., Swiderski F. (2004). Threat Modeling. Microsoft Press.

[41] Deng M., Wuyts K., Scandariato R., Preneel B., Joosen W. (2011). A privacy threat analysis framework: Supporting the elicitation and fulfillment of privacy requirements. Requir. Eng..

[42] Luna J., Suri N., Krontiris I. Privacy-by-design based on quantitative threat modeling. Proceedings of the 2012 7th International Conference on Risks and Security of Internet and Systems (CRiSIS).

[43] Mascetti S., Metoui N., Lanzi A., Bettini C. (2018). EPIC: A Methodology for Evaluating Privacy Violation Risk in Cybersecurity Systems. Trans. Data Priv..

[44] Spiekermann S., Cranor L.F. (2008). Engineering privacy. IEEE Trans. Softw. Eng..

[45] Kalloniatis C., Kavakli E., Gritzalis S. (2008). Addressing privacy requirements in system design: The PriS method. Requir. Eng..

[46] Unabhängiges Landeszentrum für Datenschutz (ULD) The Standard Data Protection Model: A Concept for Inspection and Consultation on the Basis of Unified Protection Goals 2017. https://www.datenschutzzentrum.de/uploads/sdm/SDM-MethodologyV2.0b.pdf.

[47] Commission Nationale de L’informatique et des Liberte (CNIL) Privacy Impact Assessment (PIA) Methodology—How to Carry out a PIA 2015. https://www.cnil.fr/sites/default/files/typo/document/CNIL-PIA-1-Methodology.pdf.

[48] Thorburn R., Margheri A., Paci F. Towards an integrated privacy protection framework for IoT: Contextualising regulatory requirements with industry best practices. Proceedings of the Living in the Internet of Things (IoT 2019).

[49] ResearchGate What Is the Difference between a Framework and a Model in Educational Research?. https://www.researchgate.net/post/What_is_the_difference_between_a_framework_and_a_model_in_Educational_research.

[50] Denning T., Kohno T., Levy H.M. (2013). Computer security and the modern home. Commun. ACM.

[51] Kirkham T., Armstrong D., Djemame K., Jiang M. (2014). Risk driven Smart Home resource management using cloud services. Future Gener. Comput. Syst..

[52] Jacobsson A., Boldt M., Carlsson B. (2016). A risk analysis of a smart home automation system. Future Gener. Comput. Syst..

[53] Nurse J.R., Atamli A., Martin A. (2016). Towards a usable framework for modelling security and privacy risks in the smart home. International Conference on Human Aspects of Information Security, Privacy, and Trust.

[54] Psychoula I., Chen L., Chen F. Privacy modelling and management for assisted living within smart homes. Proceedings of the 2017 IEEE 19th International Conference on e-Health Networking, Applications and Services (Healthcom).

[55] Sturgess J., Nurse J.R.C., Zhao J. A capability-oriented approach to assessing privacy risk in smart home ecosystems. Proceedings of the Living in the Internet of Things: Cybersecurity of the IoT–2018.

[56] Mohsin M., Sardar M.U., Hasan O., Anwar Z. (2017). IoTRiskAnalyzer: A probabilistic model checking based framework for formal risk analytics of the Internet of Things. IEEE Access.

[57] Ge M., Hong J.B., Guttmann W., Kim D.S. (2017). A framework for automating security analysis of the internet of things. J. Netw. Comput. Appl..

[58] National Institute of Standards and Technology NIST Privacy Framework: A Tool for Improving Privacy through Enterprise Risk Management, Version 1.0 2020. https://www.nist.gov/system/files/documents/2020/01/16/NIST%20Privacy%20Framework_V1.0.pdf.

[59] Kandasamy K., Srinivas S., Achuthan K., Rangan V.P. (2020). IoT cyber risk: A holistic analysis of cyber risk assessment frameworks, risk vectors, and risk ranking process. EURASIP J. Inf. Secur..

[60] Swiderski F., Snyder W. (2004). Threat Modeling.

[61] Shostack A. (2014). Threat Modeling: Designing for Security.

[62] Apthorpe N., Shvartzshnaider Y., Mathur A., Reisman D., Feamster N. (2018). Discovering Smart Home Internet of Things Privacy Norms Using Contextual Integrity. Proc. ACM Interact. Mob. Wearable Ubiquitous Technol..

[63] Nacci A.A., Rana V., Balaji B., Spoletini P., Gupta R., Sciuto D., Agarwal Y. (2018). BuildingRules: A Trigger-Action–Based System to Manage Complex Commercial Buildings. ACM Trans.-Cyber-Phys. Syst..

[64] Wikipedia Extended Backus–Naur Form. https://en.wikipedia.org/wiki/Extended_Backus%E2%80%93Naur_form.

[65] Jackson D. (2012). Software Abstractions: Logic, Language, and Analysis.

[66] Bugeja J., Jacobsson A., Davidsson P. A Privacy-Centered System Model for Smart Connected Homes. Proceedings of the 2020 IEEE International Conference on Pervasive Computing and Communications Workshops (PerCom Workshops).

[67] FIRST CVSS v3.1 User Guide. https://www.first.org/cvss/user-guide.

[68] NIST National Vulnerability Database. https://nvd.nist.gov/.

[69] Rosenquist M. (2009). Prioritizing information security risks with threat agent risk assessment. Intel Corp. White Pap..

[70] Bugeja J., Jacobsson A., Davidsson P. An analysis of malicious threat agents for the smart connected home. Proceedings of the 2017 IEEE International Conference on Pervasive Computing and Communications Workshops (PerCom Workshops).

[71] Barnard-Wills D. (2014). ENISA Threat Landscape and Good Practice Guide for Smart Home and Converged Media.

[72] Gray D., Allen J., Cois C., Connell A., Ebel E., Gulley W., Riley M., Stoddard R., Vaughan M., Wisniewski B.D. (2015). Improving Federal Cybersecurity Governance through Data-Driven Decision Making and Execution.

[73] Dhanjani N. (2015). Abusing the Internet of Things: Blackouts, Freakouts, and Stakeouts.

[74] Rocchetto M., Tippenhauer N.O. (2016). CPDY: Extending the Dolev-Yao attacker with physical-layer interactions. International Conference on Formal Engineering Methods.

[75] Kemmerer R.A., Porras P.A. (1991). Covert flow trees: A visual approach to analyzing covert storage channels. IEEE Trans. Softw. Eng..

[76] Lenin A., Willemson J., Sari D.P. (2014). Attacker profiling in quantitative security assessment based on attack trees. Nordic Conference on Secure IT Systems.

[77] Pieters W., Hadziosmanovic D., Lenin A., Montoya L., Willemson J. TREsPASS: Plug-and-play attacker profiles for security risk analysis (poster). Proceedings of the 35th IEEE Symposium on Security and Privacy.

[78] Arnold F., Pieters W., Stoelinga M. Quantitative penetration testing with item response theory. Proceedings of the 2013 9th International Conference on Information Assurance and Security (IAS).

[79] Mantelero A. (2016). Personal data for decisional purposes in the age of analytics: From an individual to a collective dimension of data protection. Comput. Law Secur. Rev..

[80] Department of Health Care Services List of HIPAA Identifiers. https://www.dhcs.ca.gov/dataandstats/data/Pages/ListofHIPAAIdentifiers.aspx.

[81] Martin K., Nissenbaum H. (2016). Measuring privacy: An empirical test using context to expose confounding variables. Colum. Sci. Tech. Law Rev..

[82] European Commission Guidelines on Data Protection Impact Assessment (DPIA) (wp248rev.01)—European Commission. https://ec.europa.eu/newsroom/article29/item-detail.cfm?item_id=611236.

[83] Eckhoff D., Wagner I. (2018). Privacy in the Smart City—Applications, Technologies, Challenges, and Solutions. IEEE Commun. Surv. Tutorials.

[84] Bugeja J., Jönsson D., Jacobsson A. An Investigation of Vulnerabilities in Smart Connected Cameras. Proceedings of the 2018 IEEE International Conference on Pervasive Computing and Communications Workshops (PerCom Workshops).

